# Characterization of the myocardium in the 4-chamber view using accelerated free-breathing diffusion tensor MRI

**DOI:** 10.1186/1532-429X-18-S1-P13

**Published:** 2016-01-27

**Authors:** Choukri Mekkaoui, Timothy G Reese, Himanshu Bhat, Marcel P Jackowski, David E Sosnovik

**Affiliations:** 1 grid.32224.350000000403869924Harvard Medical School - Massachusetts General Hospital, Charlestown, MA USA; 2 grid.11899.380000000419370722University of São Paulo, São Paulo, Brazil; 3 Siemens, Boston, MA USA

## Background

Diffusion Tensor MRI (DTI) of the heart *in vivo* has conventionally been performed in the short-axis of the left ventricle (LV) [1]. While this allows all three coronary territories to be seen, the short-axis acquisition orientation has some limitations. Full coverage of the LV in the short-axis requires roughly 12 to 15 slices, and accurate evaluation of the apex of the heart is often compromised. In addition, a nominal slice thickness of 8 mm is routinely used. An accelerated free-breathing DTI acquisition of the heart in its horizontal long-axis (4-chamber view) would require fewer slices (6), reduce acquisition time, and improve the characterization of the anterior and apical walls. A 4-chamber view, previously not performed, may be particularly useful when studying the remodeling of these regions.

## Methods

DTI was performed in healthy volunteers (n = 7) on a clinical 3T scanner (Siemens Skyra), with an ECG-gated STE sequence. Acquisition parameters were: FOV = 360 × 200 mm^2^, resolution 2.5 × 2.5 mm^2^, thickness = 8 mm, in-plane GRAPPA rate 2, TE = 34 ms, b-values = 0 and 500 s/mm^2^, 10 diffusion-encoding directions, 8 averages, and 12 contiguous short-axis and 6 contiguous 4-chamber slices. Rate 2 SMS excitation was followed by a blipped-CAIPI readout. A sequential acquisition of diffusion-encoding directions evenly distributes the rejections across all directions ensuring that we can select enough samples of each direction. STR was applied to reduce the misregistration resulting from respiratory motion [2]. Following STR, we utilize a novel entropy-based retrospective image selection method to reject corrupted images and maximize SNR. Mean diffusivity (MD), fractional anisotropy (FA) and helix angle (HA) values were compared between breath-hold and free-breathing.

## Results

**Figure 1:**
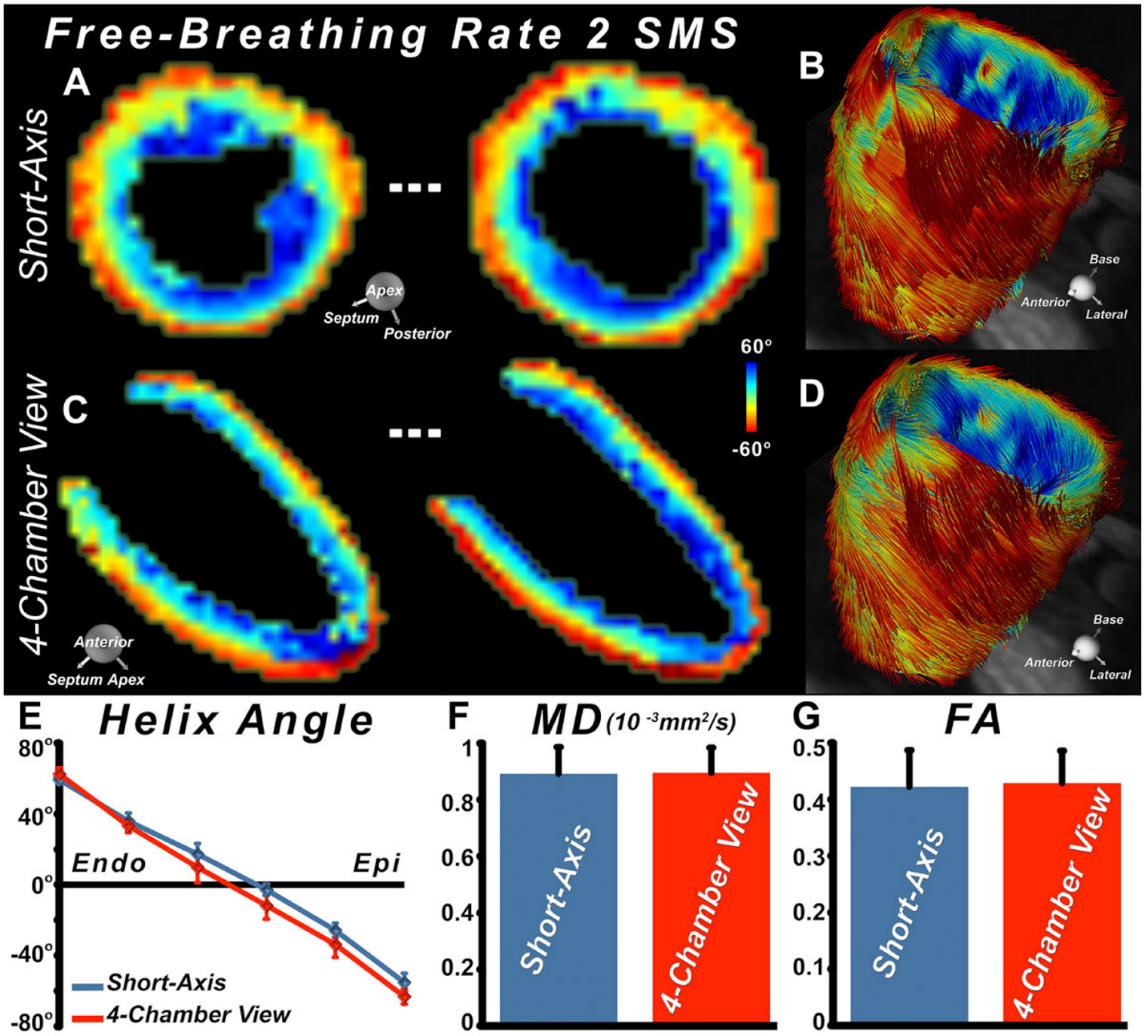
**Free-breathing DTI of the heart in the short axis and 4-chamber views**. The use of rate 2 SMS resulted in the simultaneous acquisition of two slices, and was equally effective in the short-axis (**A**) and 4-chamber (**B**) views. Consistent tractograms of the entire LV were obtained in either the short-axis (**C**) or 4-chamber views (**D**). (**E-F**) No significant differences were seen in transmural HA, MD, or FA between data acquired in the short-axis or 4-chamber views.

Accelerated free-breathing DTI acquisition of the heart could be successfully performed in the 4-chamber view. Similar HA maps and tractograms were produced from the short-axis and 4-chamber acquisitions of the LV (Figures 1A-D). There was no statistical difference in HA, MD, or FA values between short-axis and 4-chamber acquisitions of the LV (Figures 1E-G). The 4-chamber view enabled the antero-apex and true apex to be better characterized, and suggested a reduction in the number of circumferential fibers at the true apex, in addition to the two-fold reduction in total scan time.

## Conclusions

Accelerated free-breathing DTI of the human heart can be accurately performed in the 4-chamber view. This capability may be valuable in characterizing remodeling in the anterior and apical walls. Imaging the myocardium in the 4-chamber view significantly reduces scan time compared to the conventional short-axis view, which could facilitate the clinical translation of cardiac DTI.
